# Utility of [^18^F] Fluorodeoxyglucose Positron Emission Tomography/Computed Tomography Scan in Inflammatory Myopathies: Case Report and Literature Review

**DOI:** 10.1155/2018/8398453

**Published:** 2018-09-19

**Authors:** Juan Pablo Rivas-de Noriega, Javier Andrés Galnares-Olalde, Javier Zúñiga-Varga, Juan Pablo Herrera-Félix, Marco Antonio Alegría-Loyola, Alonso Turrent-Carriles

**Affiliations:** ^1^Internal Medicine Service, The American British Cowdray Medical Center IAP, Mexico City, Mexico; ^2^Nephrology Service, The American British Cowdray Medical Center IAP, Mexico City, Mexico; ^3^Neurology Service, The American British Cowdray Medical Center IAP, Mexico City, Mexico; ^4^Rheumatology Service, The American British Cowdray Medical Center IAP, Mexico City, Mexico

## Abstract

**Introduction:**

Inflammatory myopathies are a rare group of diseases characterized by proximal weakness. Incidence ranges from 7.98/million/year and prevalence at 14/100,000. The utility of [^18^F] fluorodeoxyglucose (FDG) positron emission tomography (PET)/computed tomography (CT) scan is increasing for the complementary diagnosis of myopathies.

**Case Report:**

An 84-year-old male was admitted with a history of difficulty rising from a chair and a fall. Laboratory results showed increased creatine kinase levels of more than 50 times the normal reference values. Electromyography (EMG) showed myopathic changes, and FDG-PET/CT scan showed increased FDG uptake in bilateral quadriceps. A biopsy was performed revealing lymphocytic predominant infiltrates and myonecrosis. Prednisone and intravenous immunoglobulin (IVIG) were administered with strength improvement. The patient was discharged for further follow-up.

**Discussion:**

FDG-PET/CT in inflammatory diseases has proven useful as muscle fibers have increased FDG uptake. In some cases, FDG-PET/CT is also useful in determining associated neoplastic diseases.

## 1. Introduction

Inflammatory myopathies are a rare group of diseases of unknown etiology. They are classified based on clinicopathological features in four types: dermatomyositis (DM), polymyositis (PM), necrotizing autoimmune myositis, and inclusion-body myositis [1]. Recently, the classification of other subtypes is being considered: overlap myositis, antiRO52 myopathy myositis associated with cancer, juvenile myositis, and overlap myositis. These conditions are thought to be rare and there is a lack of information regarding epidemiology. A systematic review revealed that incidence of inflammatory myopathies is 7.98/million/year and prevalence at 14/100,000 [2]. PM remains as an exclusion diagnosis, being considered the rarest of all inflammatory myopathies. Complementary imaging studies such as [^18^F] fluorodeoxyglucose (FDG) positron emission tomography (PET)/computed tomography (CT) scan can be useful in the diagnosis of inflammatory myopathies. We present a case of PM in which the FDG-PET/CT scan was useful in supporting the diagnosis.

## 2. Case Report

An 84-year-old man was admitted to our hospital complaining of muscular weakness while rising up from a chair that led to a fall. He was admitted for further studying, but his head CT and MRI did not report any abnormal structural findings. He reported a significant 10% weight loss in the past 6 months associated with decreased appetite and diminished mobility associated with progressive muscular weakness and difficulty rising from chair, with preservation of activities as combing his hair, or lifting small objects.

The physical examination was remarkable for muscular weakness with 3/5 muscle strength in the lower extremities and 4/5 of the upper extremities, confined to the proximal muscles. Tendon reflexes were diminished and the tone examination revealed mild bilateral quadriceps hypotonia and atrophy. There were no other clinical findings on the physical examination.

The patient reported a medical history of stage G4 chronic kidney disease, erythroid and megakaryocyte-predominant myelodysplastic syndrome, and high blood pressure, receiving medication with azacytidine, diltiazem, and darbepoetin. The patient did not smoke, consume alcohol, or use illicit drugs, and his family history was negative for neuromuscular diseases.

During the present admission, laboratory investigation showed an elevated creatine kinase level of up to 78,924 U/L (more than 50 times the normal reference range) and an aldolase value of 181 U/L (more than 20 times the normal reference range). Elevated serum creatinine was found (4.4 mg/dl; steady-state level 3 mg/dl), with mild hypocalcemia (7.2 mg/dl) and mild hyponatremia (130 mg/dl) with normal albumin (4 mg/dl). Thyroid hormones were normal, and cardiac enzymes were also in normal range. Coprologic examination revealed positive testing for rotavirus. Antibody testing reported negative results for antinuclear antibodies (ANAs), anti-Jo1, anti-3-hydroxy-3-methylglutaryl-coenzyme A reductase (HMGCR), anti-Mi-2, and also for antiganglioside antibodies.

The electromyography (EMG) of the upper and lower limbs showed myopathic changes in proximal muscle, with lower limb predominance, with short duration, low-amplitude polyphasic potential with no positive sharp waves, and spontaneous electrical activity.

**Figure 1:**
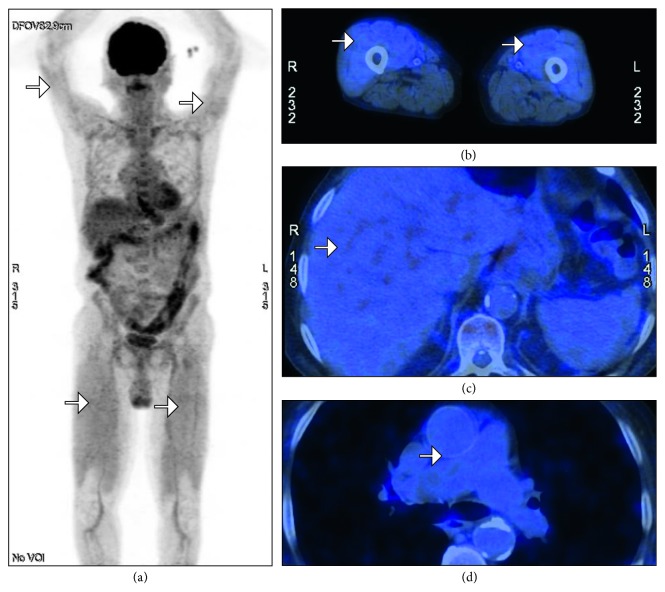
[^18^F] Fluorodeoxyglucose (FDG) positron emission tomography (PET)/computed tomography (CT). (a) Whole-body scan showing increased uptake in the proximal aspect of all limbs. (b) Proximal lower limbs that show a standardized uptake value (SUV) of 1.8 g/ml (left) and 1.6 g/ml (right); upper limb SUVs were 1.6 g/ml (left) and 1.1 g/ml (right). (c) Liver shows a more pronounced FDG uptake than all limbs with an SUV of 3.2 g/ml. (d) Mediastinal vessels were also with a more significant uptake compared to all limbs, with an SUV of 2.1 g/ml. (a–d) Areas with increased SUV signaled with white arrows.

An FDG-PET/CT scan was performed searching for neoplasia due to the patient's past history and revealed increased FDG uptake in bilateral quadriceps, without posterior compartment muscle uptake or increased metabolism in any other region of the body (Figure 1). No other location of FDG uptake was found.

**Figure 2:**
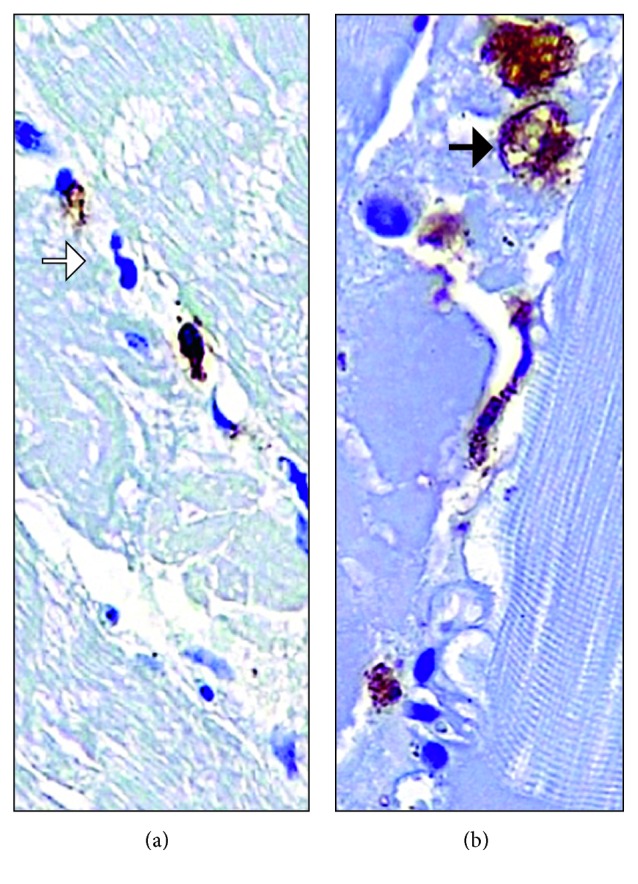
Left proximal and distal quadriceps muscle biopsy. Mild lymphohistiocytic myositis by CD8+ T cells, with severe myonecrosis (50%). The muscle fibers are healthy appearing, with some affected areas characterized by myonecrosis, phagocytosis by CD68+ cells (macrophages), and regeneration. There is no vasculitis, granulomas, eosinophilia, or thrombosis. (a) CD8+ T cell infiltrate (Gomori trichrome stain) (white arrow). (b) CD 68+ cell infiltrate (hematoxylin and eosin stain) (black arrow).

We performed an open biopsy of the left vastus lateralis muscle, which on light microscopy showed 50% myonecrosis, mild fiber atrophy, and lymphocytic infiltrate with CD8+ predominance and perivascular involvement. There were no immune deposits in the skin microscopic examination (Figure 2).

The diagnosis of PM was made and then the patient started on intravenous hydration with medium saline solution/bicarbonate for rhabdomyolysis and prednisone 0.5 mg/kg/day and intravenous immunoglobulin with a total dose of 2 g/kg distributed in 5 days, along with calcium supplementation and azacytidine. For the myelodysplastic syndrome diagnosis, steroid-sparing drugs such as azathioprine or methotrexate were not considered. The renal function of the patient improved within 5 days, with partial recovery of lower limb strength, and the patient was discharged for external consultation follow-up. After 2 months from discharge, the patient had increased muscle strength and diminished CPK levels, with low-dose prednisone as maintenance therapy.

## 3. Discussion

Inflammatory myopathies, as mentioned previously, include a wide spectrum of diseases of unknown etiology. They include PM and DM, which differ from one another in terms of cutaneous and histologic manifestations and share certain characteristics in common: slow progression, proximal and symmetric muscle weakness, creatine phosphokinase elevation (CK), myopathic pattern in EMG, as well as a mononuclear cell infiltrate by T cells and macrophages in the muscle biopsy. Both PM and DM are associated to the presence of autoantibodies like anti-Mi-2 (DM) and anti-Jo1 (DM/PM) [1, 3]. There is a strong association between DM/PM and cancer, which makes some researchers see them as paraneoplastic diseases that warrant cancer screening [4]. PM is an exclusion diagnosis once other myopathies are ruled out like DM, autoimmune necrotizing myositis, and inclusion body myositis. A muscle biopsy is required to make a definite diagnosis. In PM, there is a mononuclear cell infiltrate characterized by CD8+ T cells that surround healthy-appearing muscle fibers that overexpress MHC class I [1].

MRI has been used not only as a diagnostic method but also as a means to identify affected areas within the muscles to guide an adequate biopsy [1]. A study showed that biopsies guided by MRI were useful to find more histopathologically affected areas in the muscle; however, they found other affected areas that were not identified with MRI [5].

We researched for case series that explore the utility of FDG-PET/CT in inflammatory myopathies both in PubMed and Ovid, using the key words: “PET,” “inflammatory myopathy,” “polymyositis,” “dermatomyositis.”

FDG-PET/CT has been used since the 90s for myopathic diseases, specifically for fibromyalgia and tendinopathies. In inflammatory myopathies, there have been different case reports and case series, with polarized results. In 2011, Al-Nahhas and Jawas reported a series of 4 patients on whom FDG-PET/CT was useful to guide the muscle biopsy in order to make a diagnosis and to demonstrate resolution in only one patient [3].

Some researchers defined this as a positive result when the uptake of FDG was superior in the muscle compared with the liver and concluded that FDG-PET/CT was less sensitive for diagnosis than MRI, EMG, and muscle biopsy [6]. Tanaka et al. performed a retrospective study on 20 Japanese patients with recent diagnosis of PM/DM and the role of FDG-PET/CT in these diseases. They found that it was useful for the differentiation of PM/DM and nonmuscle diseases with an area under the curve with ROC analysis of 0.95, when using a standardized uptake value (SUV) in proximal muscles >0.83 g/ml as a cut-off. Also, there was a correlation between the SUV and muscle weakness, myositis severity, and the extent of inflammatory infiltrate in the biopsy [7].

Tateyama et al. in 2015 reported a study with 33 patients in which positive results for FDG-PET/CT reported differently than those described by Tanaka. They defined a positive result when the muscle uptake was higher than the one found in the mediastinal vessels (visually identified FDG, or vFDG). Under this concept, MRI was found to be a more sensitive study to identify affected areas within the muscle and showed two different patterns: diffuse or patches. On the other hand, FDG-PET/CT had a focal pattern in which it showed the most metabolically active muscle fibers, but had lower correlation with inflammatory infiltrate in the biopsy. There were no differences in the buttocks, whereas FDG-PET/CT was found to have more vFDG-positive results [8].

Simonsen et al. used 7 patients and found that in patients with inflammatory myopathies the SUV was superior in both limbs when compared with healthy controls with the following results: biceps (1.35 vs. 0.72 g/ml), triceps (0.91 vs. 0.44 g/ml), and quadriceps (0.84 vs. 0.62 g/ml) [9].

Inflammatory myopathies have been related to the further risk of increased malignancy incidence. According to a 2001 study published in *Lancet* by Hill et al., both PM and DM have been linked with increased cancer risk [10]. DM has a stronger association with malignancy development than PM (30% vs. 15%), with 60% diagnosed after the inflammatory myopathy diagnosis. DM was strongly associated with an increased risk in 3–5 years of the development of ovarian, lung, colorectal, and pancreatic cancer, and non-Hodgkin's lymphoma. On the other hand, polymyositis is strongly linked with lung and bladder cancers and non-Hodgkin lymphoma within the first year of diagnosis. These data support the evidence that encourages the appropriate screening for these cancers.

As described, the evidence to establish a positive result in FDG-PET/CT to diagnose inflammatory myopathies is variable. In our case, the SUV in proximal limbs was increased above the cut-off described by Tanaka et al., which is consistent with PM diagnosis. However, it did not meet the criteria of other authors, where the muscle SUV was superior to that of either the mediastinal vessels or the liver.

Our patient's diagnosis was supported by the latest European League Against Rheumatism (EULAR)/American College of Rheumatology (ACR) criteria for inflammatory myopathies, which were published in 2017 [11].

We had some limitations, such as the impossibility to obtain an MRI of the patient's lower extremities without sedation, and thus we could not compare MRI versus FDG-PET/CT findings. We also did not perform a second FDG-PET/CT since the patient had an adequate clinical response to treatment and the elevated costs of the study.

## 4. Conclusion

FDG-PET/CT has shown controversial results in terms of its utility to correlate FDG uptake to biopsy findings in affected areas. Some studies favor MRI, while others favor FDG-PET/CT. Based on Tanaka's and Simonsen's definition for positivity, our patient showed increased activity in all limbs; however, it was not superior to the SUV of the liver or mediastinal vessels as stated by Takayoshi and Tateyama, respectively. We believe that the biopsy findings did correlate with the FDG uptake on the PET-CT scan, as it was a mild inflammatory infiltrate. We did not perform an MRI on our patient so we cannot compare both results in our study. We believe PET-CT should play a more important role in inflammatory myopathies, not only because it is a full body scan but also because of the relationship with paraneoplastic syndromes.

## References

[1] Dalakas M. (2015). Inflammatory muscle diseases. *New England Journal of Medicine*.

[2] Meyer A., Meyer N., Schaeffer M., Gottenberg J.-E., Geny B., Sibilia J. (2004). Incidence and prevalence of inflammatory myopathies: a systematic review. *Rheumatology*.

[3] Al-Nahhas A., Jawad A. S. M. (2011). PET/CT imaging in inflammatory myopathies. *Annals of the New York Academy of Sciences*.

[4] Selva O´-Callaghan A., Grau J. M., Gámez-Cenzano C. (2010). Conventional cancer screening versus PET/CT in dermatomyositis/polymyositis. *American Journal of Medicine*.

[5] Studynkova T., Charvat F., Jarosova K., Vencovsky J. (2007). The role of MRI in the assessment of polymyositis and dermatomyositis. *Rheumatology*.

[6] Takayoshi O. (2012). Detection of inflammatory lesions by F-18 fluorodeoxyglucose positron emission tomography in patients with polymyositis and dermatomyositis. *Journal of Rheumatology*.

[7] Tanaka S., Ikeda K., Uchiyama K. (2013). [^18^F]FDG uptake in proximal muscles assessed by PET/CT reflects both global and local muscular inflammation and provides useful information in the management of patients with polymyositis/dermatomyositis. *Rheumatology*.

[8] Tateyama M., Fujihara K., Misu T., Arai A., Kaneta T., Aoki M. (2015). Clinical values of FDG PET in polymyositis and dermatomyositis syndromes: imaging of skeletal muscle inflammation. *BMJ Open*.

[9] Simonsen J. (2017). SPECT and PET/CT imaging in newly onset idiopathic inflammatory myopathy. *Annals of the Rheumatic Diseases*.

[10] Hill C., Zhang Y., Gigurgeisson B. (2001). Frequency of specific cancer types in dermatomyositis and polymyositis: a population-based study. *The Lancet*.

[11] Lundberg I. E., Tjärnlund A., Bottai M. (2017). European League against Rheumatism/American College of Rheumatology classification criteria for adult and juvenile idiopathic inflammatory myopathies and their major subgroups. *Annals of the Rheumatic Diseases*.

